# Facilitators and barriers to anemia prevention in the urban government childcare program for infants and young children in Peru

**DOI:** 10.1080/16549716.2025.2475580

**Published:** 2025-04-16

**Authors:** Carla Tarazona-Meza, Rosario M. Bartolini, Karina Romero, Rebecca Pradeilles, Cecilia Goya, Emily K. Rousham, Paula L. Griffiths, Hilary M. Creed-Kanashiro

**Affiliations:** a Nutrition and Dietetics, Universidad Cientifica del Sur, Lima, Perú; b Department of International Health, The Johns Hopkins Bloomberg School of Public Health, Baltimore, MD, USA; c Instituto de Investigación Nutricional, Lima, Perú; d Biomedical Research Unit. A.B. PRISMA, Lima, Peru; e UMR MoISA (Montpellier Interdisciplinary Centre on Sustainable Agri-Food Systems), (Univ Montpellier, CIRAD, CIHEAM-IAMM, INRAE, Institut Agro, IRD), Montpellier, France; f Centre for Global Health and Human Development, School of Sport, Exercise and Health Sciences, Loughborough University, Loughborough, UK; g School of Clinical Medicine, University of the Witwatersrand, Johannesburg, South Africa

**Keywords:** Anemia, complementary feeding, animal source foods, iron, infants, young children, day-care service, qualitative, LMIC

## Abstract

**Background:**

Anemia is a major public health problem in children under 2 years old in Peru and other low- and middle-income countries.

**Objectives:**

We aimed to explore facilitators and barriers to anemia prevention through feeding strategies in the Cuna Mas day-care centers in urban Peru among infants and young children (IYC) aged 6–23 months.

**Methods:**

Qualitative research in day-care services (*n* = 14) in Peru. We conducted direct observations and semi-structured interviews about consumption of animal source foods (ASF) and iron supplementation with day-care staff (technical managers, nursery caregivers and ‘mother guides’) and caregivers of IYC. We applied a grounded approach to data analysis.

**Results:**

Cuna Mas staff facilitated ASF consumption by introducing small portions of iron-rich foods and serving organ meats in tasty stews. Staff also avoided giving carbohydrate-rich foods to IYC prior to giving organ meats. Staff facilitated giving daily iron supplement by using standardized and personalized strategies, such as using a timed reminder, praising and cheering a child or giving supplements whilst washing a child’s hands and face. These strategies were also shared with primary caregivers to use at home. Some barriers reported by caregivers included difficulties in accessing and preparing specific ASF at home.

**Conclusion:**

The Peruvian Cuna Mas complementary feeding program facilitated consumption of iron-rich ASF and iron supplements through a range of strategies which they then shared with caregivers for implementation at home. These institutional behavioral change initiatives could be replicated in other settings whilst considering the facilitators and barriers identified in this study.

## Paper context

- **Main findings**: Anemia remains a major public health concern for infants and young children in many low- and middle-income countries including Peru. We conducted qualitative research into the program for anemia prevention and treatment in the Cuna Mas day-care service in urban Peru. Our findings show multi-sectoral integration and grass roots community structures
- **Added knowledge**: We identified facilitators and barriers on the consumption of iron-rich animal source foods and iron supplementation.
- **Global health impact for policy and action**: The approaches reported in our study can inform nutrition-based interventions in childcare settings.

## Background

Infant and young child nutrition is of central importance for healthy physical and cognitive development. This period of life also offers a window of opportunity to prevent multiple forms of malnutrition including micronutrient deficiencies. The World Health Organization (WHO) recommends the initiation of complementary feeding at 6 months of age, introducing meals with a variety of foods, without added sugar or salt, and to gradually increase the meal frequency and quantity with age to prevent malnutrition [1]. In addition, the WHO guidelines recommend daily iron supplementation for infants and young children (IYC), 6–23 months, living in areas where anemia prevalence is 40% or greater in this age group [2].

Anemia represents a major public health concern in pre-school children with a prevalence of 40% globally with serious effects on cognition and other functions throughout the life course [3]. Anemia is primarily characterized by low hemoglobin concentration in red blood cells. One of the leading causes of anemia is the imbalanced intake and absorption of micronutrients, especially iron [4]. However, recurrent infections, lack of access to sanitation and healthcare services as well as other socioeconomic factors remain important underlying causes of anemia in many low- and middle-income countries (LMICs), including Peru [5–8].

In 2016, the prevalence of anemia in Peru was estimated at 33.3% in children under 5 years and 43.6% in children under 3 years of age [9]. As a result, a multi-sectoral national plan to combat and prevent anemia in children under 3 years was established in April 2017, focused on increasing the intake of iron-rich animal source foods (ASF) and iron supplementation in target groups [5]. Iron-rich ASF include organ meats such as chicken liver, beef liver, spleen and chicken heart as well as chicken blood. National policies were developed to tackle anemia through multi-faceted approaches in different government institutions. One component of this multi-sectoral plan is the Cuna Mas child national program from the Ministry of Development and Social Inclusion (MIDIS). This national program has focused on early childhood development, providing day-care services in low-resource urban settings, and household visits in rural areas [10,11]. The Cuna Mas day-care program targets the population living in poverty or extreme poverty and aims to provide integrated care for healthy growth and development, where nutrition is valued as one of the pillars along with cognitive, emotional and motor development [12,13].

Various interventions have been implemented focused on anemia prevention and treatment in children from LMICs and several factors may influence their results. In Ghana, a qualitative assessment reported that local traditions led to the rejection of certain iron-rich ASF and limitations on adopting the education intervention recommendations in low-resource households [14]. In Peru, mothers of IYC diagnosed with anemia attending a primary health care center reported hesitancy about iron supplementation and its benefits, suggesting that a balanced diet should suffice to treat anemia [15].

The potential effect of the multi-sectoral national plan to combat anemia in children between 2–4 years from Madre de Dios, an Amazonian region, was estimated to increase mean hemoglobin concentration by 0.89 g/dL (0.45–1.32) with a greater effect among those living in indigenous communities with less access to health interventions [16]. Evaluation of the Cuna Mas program has shown promising results on child development but the impact on anemia prevention and treatment remains unclear [13]. Likewise, the process of implementing the multi-sectoral national plan in urban day-care centers has not been reported, even though day-care services represent potentially important places to promote healthy nutrition in IYC [17,18]. We consider that the potential strengths and limitations of the Cuna Mas program on improving nutritional knowledge and practices relating to consumption of iron-rich foods and iron supplements have not been investigated qualitatively, hence the rationale for this study.

In this study, we aimed to explore facilitators and barriers to implementing anemia prevention programs through IYC feeding strategies in the Cuna Mas day-care centers in two peri-urban settings of Peru. Specifically, we aimed to gain insights into facilitators and barriers to consumption of iron-rich ASF and iron supplements from the perspective of the different actors involved.

## Methods

### Qualitative approach and research paradigm

This qualitative study followed an ethnographic and grounded theory approach using in-depth interviews to understand the narratives, perceptions and reflections underlying anemia prevention approaches. This was supplemented with direct observations of feeding and iron supplementation practices within the day-care centers. This approach also triangulated the perspectives of the staff who provided the day-care service and primary caregivers of IYC, while comparing data across the different urban day-care service centers.

This qualitative study was part of a larger interdisciplinary project PERUSANO (Peru – Estrategias y opoRtUnidad: Sin Anemia Ni Obesidad) which aimed to address multiple forms of malnutrition, particularly stunting, anemia, and risk of overweight/obesity in peri-urban Peruvian IYC aged 6–23 months [19].

### Context

The study was conducted in Cuna Mas day-care centers in two peri-urban settings of Peru: Manchay, in Pachacamac District, in the outskirts of Metropolitan Lima, and Huanuco district at 1,880 m above sea level in the Andean highlands. These two low-income peri-urban communities are settlements of internal and regional migration, where the housing areas of more recent migrants lack basic services such as potable water and sewage services. We chose peri-urban communities from these two cities because Lima is the largest metropolitan center in the country, housing approximately 30% of the national population, and Huanuco is more typical of the smaller cities reflecting the growing population of cities in outlying areas of the country [20].

In urban settings, the Cuna Mas program works through day-care services due to the population density in cities, where they can bring enough IYC together to be viable. The urban day-care service runs from 8 am to 4 pm and has three different types of facilities depending on their infrastructure, personnel and child provision: day-care households, day-care centers, and comprehensive infant care centers. The urban day-care centers provide food three times a day to children aged 6–36 months consisting of age-appropriate food and meeting 70% to 100% of energy, protein, zinc, calcium and iron daily recommended intakes [13].

The actors involved in the Cuna Mas day-care centers for IYC are: i) district technical managers, ii) nursery caregiver volunteers, and iii) mother guides. The district Cuna Mas technical managers, primarily women, have a university degree in education, health or social sciences or higher technical training with at least 1 year of work experience in similar programs. They have responsibility for overseeing the activities and administration of 4–6 Cuna Mas facilities [13]. Each day-care center has nursery caregiver volunteers; women who are at least 21 years old, with ideally a completed secondary education, who usually live in the same community/district. Overall, there is one nursery caregiver volunteer for every 3 to 4 children aged 6–18 months old, and for every 8 children aged 19–36 months old. They are responsible for playing and taking care of the children, including feeding them at meal times [21]. The mother guides are women who are at least 21 years old with previous community work experience. As well as their coaching and supervisory role within the day-care facilities, they carry out home visits to households attending the Cuna Mas periodically to support and promote feeding practices to prevent anemia. The nursery caregiver volunteers and mother guides have constant interaction with the primary caregivers of the children [21].

### Sampling strategy

The study was conducted in 14 of the 16 Cuna Mas day-care facilities identified within the PERUSANO study areas: eight in Huanuco and eight in Manchay. Cuna Mas centers were purposively selected to represent the diversity in the type of facilities and the number of IYC enrolled. We did not include centers with few or no children in the target age range.

The Cuna Mas day-care staff were recruited purposively based on i) having responsibility for children aged 6–23 months, ii) availability for interview and iii) representing the different roles outlined above. We also recruited primary caregivers, defined as the person who dropped off and picked-up their children at the day-care facilities. Primary caregivers were selected through opportunistic sampling when they dropped-off or picked-up their child from the day-care facilities [22,23].

### Data collection methods

Data collection for this study was conducted between the end of 2019 and the beginning of 2020, prior to the COVID-19 pandemic lockdown. The study was carried out by a multidisciplinary team with previous experience in qualitative research from the Instituto de Investigación Nutricional (IIN) in Peru and Loughborough University, UK.

The data collection field team was formed of four women anthropologists, two allocated to each field site, with university bachelor’s degree. The field team coordinator had a master’s degree and extensive previous experience in qualitative techniques and fieldwork in Peru. A month-long training period was held with the field team to focus on the underlying research aims, qualitative data collection techniques and background on the national day-care program. All data collection guides were refined during the training sessions and piloting.

### Data collection instruments

The semi-structured guides were developed by the multidisciplinary team in Spanish and translated to English for the purposes of refining questions and themes among the full research team. All changes were incorporated into the final version in Spanish.

#### Interviews

The semi-structured guides for in-depth interviews included the following main topics: i) characterization and composition of food and meals, ii) perceptions of IYC nutritional status, iii) facilitators and difficulties with iron supplementation and reflections about the quality of the service, iv) characteristics of care and feeding methods, v) communication, and vi) interactions with users.

While we used the semi-structured guide for these interviews, emerging themes were allowed and included in subsequent interviews. Likewise, any emerging actors in large day-care facilities were identified and included in the protocol. Data collection was overseen by the anthropologist with the most expertise in qualitative research in each site.

Semi-structured in-depth interviews with the day-care staff were carried out at the day-care centers and had a mean duration of 40 min. Short interviews were conducted with primary caregivers outside the day-care center at the time of collecting or dropping off their IYC and lasted from 15 to 20 min. The first interviews were conducted with the Cuna Mas technical managers in the study sites. After this, the technical managers introduced the field anthropologists to staff in each of the selected day-care centers to arrange a date for the first direct observation.

#### Direct observations

The principal themes in the guides for the direct observations were focused on i) feeding times, ii) intake of iron-supplements, and iii) hygiene events.

Direct observations were carried out by one anthropologist for one day at each day-care center from 8 am to 2 pm or 3pm. The field team anthropologist was allowed to participate in general activities but did not take part in meal time feeding or iron supplementation. Some participation in general activities enhanced the trust between the nursery staff and anthropologists, which led to a greater rapport for the in-depth interviews. Observation data were recorded using key words about daily events, for rapid recording in field notebooks. On the same day as the visit, the anthropologists transcribed their notes into fully written accounts of all the observed events.

### Data analysis

Interviews were audio-recorded and transcribed in Spanish verbatim by the four anthropologists who conducted them. Interviews transcripts and direct observation reports were coded using NVivo 12 software. A grounded theory approach to analysis was used throughout the research [24]. The semi-structured guides for the direct observations and interviews provided the initial framework for themes. The field team coordinator was responsible for the quality control of interview transcripts and direct observation reports.

The analysis was conducted by the four field anthropologists and one of the researchers (RB), verifying and discussing the codes and outputs from NVivo whilst allowing for emerging themes. The thematic outputs from transcripts of the direct observations and interviews were read and interpreted by three other researchers from the interdisciplinary team following a thematic analysis, comparing the results between study sites and identifying differences and similarities relating to ASF consumption and iron supplementation. Further discussion between all the investigators was used to triangulate data from interviews and observations and identify prior themes as well as newly emerging themes.

Data were coded according to the major pre-defined themes for direct observations (feeding times, intake of iron-supplements, hygiene events) and interviews (characterization and composition of food and meals, perception of the nutritional status of the child, facilitators and difficulties with iron supplementation and reflections about the quality of the service, characteristics of care, feeding methods, communication, and interactions with users) as well as newly emerging sub-themes relating to facilitators and barriers to the iron-rich food intake and iron supplementation.

All analyses were conducted in Spanish and then the quotes were translated to English. Other than the city location (Lima or Huanuco), the identity of facilities and participants were anonymized for all observations, recordings, transcripts and quotes.

## Results

We conducted eight direct observations in Huanuco and six in Lima within the 14 participating Cuna Mas day-care facilities. We conducted 14 semi-structured interviews (six in Huanuco, eight in Lima) with staff of the day-care facilities (seven interviews with nursery caregivers; four with technical managers, three with mother guides); and 22 short interviews with primary caregivers (12 in Huanuco, 10 in Lima). The breakdown of direct observations and interviews within specific staff roles is provided in Table 1.

**Table 1.** Summary of the sample and data collection methods.

		Number (n) of interviews/observations	
Participants	Data collection method	Huanuco	Lima
Day-care facilities	Direct observations	8	6
Day-care staff			
District technical manager	Semi-structured interviews	2	2
Nursery caregiver volunteer	Semi-structured interviews	2	5
Mother guides	Semi-structured interviews	2	1
Primary caregivers			
Primary caregiver	Short interviews	12	10

We report the findings relating to the two major themes: i) consumption of iron-rich ASFs and ii) provision of iron supplementation. The identified subthemes are then presented as facilitators and barriers for the day-care staff and primary caregivers (Table 2).

**Table 2.** Summary of themes and subthemes relating to facilitators and barriers.

Themes	Facilitators or barriers	Subthemes
Consumption of iron-rich animal source foods	Facilitators	Enhancing the consumption of iron-rich animal source foods in meals
		Day-care staff activities to reinforce messages and strategies to primary caregivers
	Barriers	Initial resistance to iron-rich animal source foods among IYC
		Primary caregivers’ perceptions about iron-rich animal source foods
		Primary caregivers’ provision of iron-rich animal source foods in the home
		Primary caregivers’ perceptions about iron-rich ASF offered at the day-care centers
		Food planning and meals preparation at the day-care service
Iron supplementation	Facilitators	Iron supplements kept at the day-care facilities
		Enhancing the daily consumption of iron supplements
		Monitoring of iron supplementation in the household

### Consumption of iron-rich ASFs

A summary of the findings with supplemental quotes regarding facilitators of iron-rich ASF consumption among IYC is shown in Table 3.

**Table 3.** Facilitators of consumption of iron-rich animal source foods in the day-care service.

Subthemes	Quotes
Enhancing the consumption of iron-rich animal source foods in meals	**REF1:** ’… liver, I have always made it in soup, with vegetables, with giblets, but not there. I have seen that they (Cuna Mas staff) make it with milk, and my child likes it’. **[Primary caregiver]** *What happened to the sangrecita (chicken’s blood) and the bofe (blood and beef lung)?* −Child didn’t eat it. I did prepare it, but he didn’t eat it. I used to make it in soup. I would give him the ram’s spleen, scrape it, give it to him in soup or give him some water, he didn’t drink it. But if you mixed it in his mazamorra (porridge) or in something she was eating, she would eat it. *Do you remember how they gave it to her at Cuna Mas?* -‘They always gave it to her in the mazamorrita (porridge). I remember they gave her that. That’s how she got her mazamorrita. Fried liver or blood, that’s how she got it, but she ate it well. I fry the liver, she doesn’t eat it. That’s why I tell you, maybe I give it too fried or badly fried. I don’t know how you eat, I ask her (her daughter); ‘I like it like this’ she tells me. But then I give it to her, she doesn’t eat it’. **[Primary caregiver]** **REF2:** ‘I rather see that here [in the Cuna Mas] the children like to eat the liver or blood ´seasoned´. When it is mixed with carbs, I observe that children don’t eat them… So, here, you will see that the first thing children grab is their meat – liver, blood – whatever. I say, ‘how well they eat the blood’, they grab it, they eat it first, ‘well, that’s the most important thing’ **[District technical manager]**
Day-care staff activities to reinforce messages and strategies to primary caregivers	**REF3:** *Have they taught you here at Cuna Mas, for example, during the demonstration workshops on nutrition how to cook iron-rich foods of animal origin?* -Yes, that has been pretty helpful to replicate those meals at home.” **[Primary caregiver]** **REF4:** ‘*From what was explained to you about feeding your children, from what you were shown, what could you put into practice at home, any preparation?* -Yes, the s*angrecita* (blood) because we had never eaten it at home … *How did they tell you that you had to prepare it?* -They gave me a recipe booklet. There were recipes but basically it was sangrecita (blood) because we had never prepared it at home. *Did you try to make mazamorra de sangrecita (porridge of blood) with rice at home?* No. *Would you try?* Sure, I would try, but since I don’t know how to do it, I haven’t done it yet’ **[Primary caregiver]**

#### Facilitators

##### Enhancing the consumption of iron-rich ASFs in meals

The Cuna Mas staff designed and implemented successful ways of enhancing the consumption of iron-rich foods of animal origin in the daily meals of children. These were also observed by the research field team. Firstly, the staff prioritized the consumption of iron-rich ASF before offering carbohydrate-rich foods to avoid early satiety. ASF intake was facilitated by not mixing carbohydrates, typically rice and potatoes, with ASF and avoiding large mouthfuls of iron-rich ASF which are more difficult for IYC to swallow. The second facilitator for this strategy that they reported was to avoid ‘dry’ meals and instead provide juicy meals containing iron-rich ASF. Juicy foods that are not so dry were more acceptable to IYC. Iron-rich ASF (e.g. liver, blood) were well tolerated when mixed in traditional porridges (‘mazamorras’), also as small pieces in stews (‘guisos’).

***What do you think makes it easier to feed children, i.e. what makes it easier for you to get them to eat?***

*The stews.*

***Food with stews, that’s what they like the most?***

*-Yes, stews, stew, all kinds of stews, that’s what they eat. Children want juicy meals…*

**[Nursery caregiver volunteer]**

##### Day-care staff activities to reinforce messages and strategies to primary caregivers

The day-care staff reported that they continually explained and raised awareness among the primary caregivers about the reasons for, and benefits of, consuming iron-rich ASF as a strategy of the Cuna Mas program to reduce childhood anemia. In other words, the process of Cuna Mas staff raising awareness among caregivers, with regularity and consistency, led to greater acceptance of the importance of iron-rich ASF among caregivers. In addition, several primary caregivers highlighted the importance of the demonstration sessions provided by the Cuna Mas staff and health centers that showed how to prepare and cook iron-rich ASF, as well as suggesting new recipes for IYC. Primary caregivers highlighted the role of the day-care service program in providing ‘*more nutritious food, according to their children´s age needs*’.

### Barriers

Our study identified five main sub-themes relating to barriers to the consumption of iron-rich ASF. A summary of the quotes on this theme is shown in Table 4.

**Table 4.** Barriers and challenges on the consumption of iron-rich animal source foods.

Subthemes	Quotes
Initial resistance to iron-rich animal source foods among infants and young children	**REF1:** ‘(…)*when they receive blood, liver, spleen, do the children accept it well, do they like it*? -Well, the blood [sangrecita] is frequently accepted without major issues. But, regarding the liver, I think that sometimes the Cuna Mas program makes a bad mix of liver and legumes because both are very dry, difficult to eat quickly by children(…)’ **[Nursery caregiver volunteer]** **REF2:** ‘No, my little boy, for example, for the first weeks, almost two weeks, well, it affected his stomach a little bit, because of the same iron, well, the same food is different at home, well, as you don’t know how to make stew, well, pure chicken, chicken at home. So, I think that, that change caused my son a stomach infection, but later he recovered, he adapted quite a bit’ **[Primary caregiver]** **REF3**: ‘Liver, yes, but it caused a reaction; even in the crib he had a reaction because he had some pimples, but now, he has adapted, he doesn’t get them anymore. *How long did it go on for?* -It was like that for about 5 days, but now it is drying up, right? But every time I gave him liver he had that reaction, but not now’. **[Primary caregiver]**
Primary caregivers’ perceptions about iron-rich animal source foods	**REF4:** ‘There are others who (say) they are shocked, others that they should not give it like that, that it is ugly. When the blood comes…for example, they make porridge of blood, no, they don’t like it, ‘How can they give that? My child will get sick,’ they say (…) others say ‘no, because he is weak’, others say ‘no, it makes him sick to his stomach’ **[Nursery caregiver volunteer]** **REF5:** ‘*What do you think of these foods, the liver, the blood, what do you think they are good for?* -For anemia, hemoglobin, to combat anemia. *In what cases could these types of foods be harmful for a child? Do you think they could be harmful?* −I have heard that it depends on what type of blood they use. There are some who are shocked by pig’s blood. There is a mom who told me that if it is pig’s blood, don’t give it to my son because it shocks his stomach, he will give it all back to me.’ **[Nursery caregiver volunteer]** **REF6: ‘**I had children who did not consume blood because of religion. They didn’t consume blood, they didn’t want to. **[District technical manager]**
Primary caregivers’ provision of iron-rich animal source foods in the home	**REF7:** ‘*And all the recommendations that has been given to you, do you practice them at home?* -Yes (…) sometimes I don’t, not when there is a lack of money, I can’t, because I must have; sometimes I must feed her what there is, too (…). For her (…) I always add her spleen for her (…), with her chicken chili, but she must have a little piece of liver for her. *Isn’t it easy to get spleen in the markets here in Huanuco, or does it run out quickly?* -Yes, it runs out quickly. (…) I have to go to the market early in the morning to find it. Sometimes I can’t because my son is studying (he attends the morning shift) (…) The cost is seven to eight soles, each spleen. *And can you only find it in the market or can you get it elsewhere?* -No, only at the market and at the slaughterhouse where the cattle are slaughtered (…) [**Primary caregiver]**

#### Initial resistance to iron-rich ASFs among IYC

Nursery caregivers and primary caregivers reported that IYC had a lack of appetite, would reject meals and food and/or had short-term digestive problems during the first 2 weeks of starting at the day-care centers. Some primary caregivers stated that these iron-rich ASF are unpleasant for their children (e.g. ‘*the blood shocks their stomach; the liver gives them skin allergies’*). However, the nursery caregivers mentioned that they did not observe any actual sickness after the children ate meals containing blood, or other ASFs, at the day-care centers, rather there was some initial hesitance to consuming such foods. Similarly, both the nursery caregivers and primary caregivers noticed that the IYC adapted to meals containing iron-rich ASF after this initial period of resistance. The process of IYC adapting to the new foods and meals was facilitated by using the strategies outlined in the previous section.

#### Primary caregivers’ perceptions about iron-rich ASFs

The mother guides reported that primary caregivers expressed concerns about preparing iron-rich ASF in their homes which was a barrier to consumption. In short interviews, the primary caregivers explained their concerns about iron-rich ASF which related to three main factors: i) palatability (*‘those are ugly tasting foods’*), ii) fear about possible adverse health effects of consuming such foods (‘*it shocks the stomach*’, ‘*pork blood meals would upset the children*’, or ‘*blood is not good for a younger child, it shocks them, it´s bad for their stomach*’) and iii) religious beliefs, specifically about blood consumption, which the Cuna Mas staff also reported about some primary caregivers. Some primary caregivers disagreed with the Cuna Mas staff, reporting that they [Cuna Mas staff] ‘*don’t understand why pork blood is bad for children*’ and that meals with liver caused ‘*allergy*’ (skin rashes) in their child.

#### Primary caregivers’ provision of iron-rich ASFs in the home

Primary caregivers reported that the nutritional recommendations and advice provided at Cuna Mas were, in general, easy to implement at home. Furthermore, primary caregivers acknowledged the importance of prioritizing the regular consumption of iron-rich ASF to prevent childhood anemia, as well as the overall well-being of their children. However, when asked specifically about giving the recommended iron-rich foods of animal origin to their IYC at home, some mothers reported difficulties and/or lack of knowledge of how to prepare these foods.

The barriers to implementing these practices related to economic difficulties, limited availability of certain foods, or difficulties in accessing these foods.

***Have you tried, for example, to cook blood and spleen at home?** Yes, I’ve looked, but it’s very difficult here, isn’t it, to find, because there’s a man who only brings it on Saturday, and I did find some. I found it [blood] black and dirty; then I said, “I won’t be able to feed that to my son”, so I said “no”. What I found instead was chicken liver, that’s what I’m giving him, that’s what he eats, that’s what he takes from me with peace of mind*

**[Primary caregiver]**

Primary caregivers in Huanuco city tended to report more problems relating to access and availability of iron-rich ASF than their counterparts in Manchay. Some iron-rich ASF were particularly difficult to obtain in Huanuco, such as spleen or ‘sangrecita’ (blood), because it was necessary to go to the market or to the slaughterhouse in the early hours of the morning to buy them. In Manchay, caregivers reported that these foods were available, but sometimes sold in poor condition. This barrier was overcome by the availability of other ASF which caregivers could buy.

#### Primary caregivers’ perceptions about meals with iron-rich ASF offered at the day-care centers

Primary caregivers suggested that IYC may become bored of meals provided by the day-care centers if the dishes lacked diversity or were ‘too dry’. In addition, they reported that some menus had a low variety of vegetables and did not include fish. Thus, they suggested adding more ‘color’ and flavor to the meals, providing variety by including dishes and drinks with different tastes, even some fried foods. Stews were the most common main meals in the Cuna Mas facilities, so primary caregivers suggested varying the menus to include ‘grilled or fried’ foods which have more taste and will stop children from getting bored. Beef was the least consumed ASF because it is a little tougher than other meats, thus the caregivers suggested making dishes such as ground beef, tortillas or mixing beef with other ingredients to make it more tender.

#### Food planning and meals preparation at the day-care service

Based on the primary caregivers’ observations and suggestions, the technical managers for day-care centers coordinated with the kitchen partners, who prepared meals, to improve the cooking of the vegetables and make certain foods softer or juicier. Nevertheless, the day-care staff pointed out the lack of a formal channel or standard procedure to pass on feedback from the primary caregivers on food planning and cooking to the MIDIS local authorities who led the program. Since meal planning includes exact quantities for each ingredient in every meal which the catering staff had to follow, it was difficult to change the quantity or quality of the ingredients to provide more flavor, color and diversity according to the suggestions of the day-care staff and primary caregivers.

### Iron supplementation

In addition to the consumption of iron-rich animal food sources, iron supplementation to prevent anemia was another mandatory activity within day-care services. A summary of the findings with supplemental quotes about the facilitators for iron supplementation is reported in Table 5.

**Table 5.** Facilitators of iron supplementation to prevent anemia in children from the day-care program.

Subthemes	Quotes
Iron supplement stock at the day-care facilities	**REF1:** ‘ … We ask for the iron supplement, we ask whether the child is still consuming it or not; if the child is in an age to consume the micronutrient, then, they have to consume it. If the child is not consuming, not willing to, well, unfortunately -and this is very sad-, the child cannot take part in Cuna Mas (…) *Is this a requirement? (…) that the child is consuming iron supplements*. -Exactly, because this year, what we must prevent is anemia; So, our first thing where we have to work hard on, it is anemia. And mommies must understand us’ **[Mother Guide]** **REF2:** ‘*Regarding the iron supplementation, in this Cuna Mas center, since when has the iron supplementation (iron supplement) been administered**?*** -Mostly when children enter (to the Cuna Mas program) with anemia, we administer the iron supplementation. Previously there were ‘chispitas’ multi-micronutrients (MMN) and sulfate (iron sulfate), the children with anemia took sulfate, the ones who did not (have anemia) took ‘chispitas’ multi-micronutrients, now they have changed this (around a month, two months ago), and now everything administered is iron supplement, as a preventive and for those with anemia’ [**Nursery caregiver volunteer]**
Enhancing the daily consumption of iron supplements	**REF3:** ‘In the training they told us that first, to prevent anemia, they are going to give micronutrients or sulfate. At 11:00 (a.m.) sharp, the children must be taking their sulfate and those children with treatment will also receive the iron supplements but with a prescription to revise the number of drops that I have to give the child, taking into consideration their weight, and with the mother´s consent, who signs the informed consent document’ [**Nursery caregiver volunteer]** **REF4:** ‘*Is the iron supplement administration always accompanied by a drink that is water?* -Yes, water. *And why is the iron supplement given to them like this, with a sip of water?* -Big question; the truth is that we received the order to provide a citrus fruit drink in addition to the iron supplement liquid, but I don’t know why we are using water alone. *Is that what they have indicated?* -Yes, but sometimes, I think that the citrus fruit must be freshly prepared, and we don’t have this available, we can’t do that, that’s why we just provide a bit of water as a replacement.’ [**Nursery caregiver volunteer]**

### Facilitators

#### Iron supplement stock at the day-care facilities

The iron supplements used in all of the included Cuna Mas facilities were ferrous sulfate in syrup and drops, and iron polymaltose. Primary caregivers of IYC had to provide their child’s iron supplementation prescription, whether to prevent or treat anemia, to the day-care service staff as a requirement of using the service. The primary caregivers received their child’s iron supplements from their local health center and brought them to the day-care facilities. More recently, the mother guides have been authorized to request the iron supplements directly from the health facilities in coordination with the primary caregiver and day-care staff. This new process is perceived as an improvement by all the actors involved, including primary caregivers. This ensures that the weekly iron supplements for each child are held at the Cuna Mas during the week. At the weekend, the iron supplement bottles are returned to the primary caregivers who must administer it to their child. Every Monday, nursery caregivers verify that the child has their iron supplements for the week. If not, the primary caregiver is asked to return home to bring them immediately. In one health facility, iron supplement bottles were organized in such way that each child has a bottle at home and another in the day-care facility. This strategy was perceived by primary caregivers as a facilitator as it provided them with additional support.

*We ask for the iron supplement, we ask whether the child is still consuming it or not; if the child is at an age to consume the micronutrient, then, they have to consume it. If the child is not consuming, not willing to, well, unfortunately (and this is very sad), the child cannot take part in Cuna Mas*

***Is this a requirement?…that the child is consuming iron supplements**.*

*-Exactly, because this year, what we must prevent is anemia; So, our first thing where we have to work hard on, it is anemia. And mommies must understand us”*

**[Mother Guide]**

#### Enhancing the daily consumption of iron supplements

Nursery caregiver volunteers administered iron supplements on weekdays at the day-care center, following the standardized healthcare prescription and protocols. Observations showed that iron supplement bottles were kept in a designated place (e.g. baskets with lid) and labeled for each child. The staff used reminders such as an alarm set between 10:30 and 11 am, to start preparing the children for their daily iron supplement. They followed the medical guidance to provide the supplements at least 1 h before or after meals (e.g. on an empty stomach to enhance absorption). The nursery caregivers asked the children to line up starting with the older and/or most receptive ones. Personalized strategies were also developed for individual IYC in the day-care facilities. These facilitators were implemented especially for those who were new to the facility or those that did not readily accept the supplement. Examples of personalized strategies were administering the iron supplements while playing or bringing the child to wash their hands and face before taking the supplement or giving the child water before or after taking ferrous sulfate to ensure consumption. Nursery caregiver volunteers also cheered and congratulated the child after consuming the iron supplement. The day-care staff acknowledged that both actors, the child and the nursery caregiver volunteer, had to adapt to each specific situation using individualized strategies. For example, if the child did not consume the entire iron supplement dose, the nursery caregiver waited and offered it once or twice more. Furthermore, nursery caregivers also brushed the children’s teeth after taking the iron supplement to remove the taste and reduce teeth staining. This entire process of iron supplement intake lasted approximately 6 min per child. When a child rejected the iron supplement, various personalized strategies were applied (e.g. little airplane distraction, imitating drinking, giving it to another child first, putting it in a glass of water). As a final resort, nursery caregivers would open a child’s mouth to ensure consumption, in a non-responsive feeding manner, emphasizing that it is mandatory to provide the supplement.

The day-care staff were aware of the health center recommendations to consume the iron supplement with Vitamin C rich foods, such as citrus fruits, but they decided to give it with water due to hygiene considerations, lack of time, and the need to adhere to the scheduled provision of foods and drinks. Soup spoons or measuring spoons were used to administer the syrups.

***Is the iron supplement administration always accompanied by a drink that is water?*** Yes, water.

***And why is the iron supplement given to them like this, with a bit of water?***

(…) I think that the citrus fruit has to be freshly prepared, and we don’t have this available, we can’t do that, that’s why we just provide a sip of water as a replacement.

**[Nursery caregiver volunteer]**

#### Monitoring of iron supplementation in the household

Iron supplementation during weekends occurred in the child’s household, and it was promoted and closely monitored by the day-care mother guides, who also provided guidance when adverse effects, such as constipation or stained teeth, were reported. In addition, they also supervised and provided guidance if a child was refusing to take the supplements. The mother guide visited the healthcare centers whenever the child needed iron supplements. Mother guides also visited households of children attending day-care centers to reinforce the main messages about infant and young child nutrition and development to the primary caregivers. Mother guides also identified the potential needs of primary caregivers at the household level that could be incorporated into the future cooking demonstrations and educational sessions. The primary caregivers considered that all of these various strategies were important facilitators that helped their children to consume iron supplements.

## Discussion

### Summary of findings

Our findings elucidate the facilitators and barriers to promoting the consumption of iron-rich ASF and iron supplements in children aged 6–23 months within the Cuna Mas day-care centers in two urban regions of Peru.

From primary caregivers, some barriers and restrictions related to the availability, affordability, use and storage of some iron-rich ASF which limited their ability to implement certain practices and recommendations from the day-care service. However, primary caregivers overcame this barrier by seeking more accessible ASF. Primary caregivers highlighted the importance of the cooking demonstrations that took place during the mother guide home visits and other support mechanisms provided by the mother guides. These findings are consistent with other studies about the effectiveness of community-based interventions where improving cooking skills had a positive impact on diet and health outcomes, providing more benefits to vulnerable population in low-resource settings [25–27]. However, previous evidence also indicates that nutrition education alone may not be enough to achieve behavior change, particularly in low-income communities with food insecurity risk, hence other simultaneous strategies are needed [28]. Therefore, the meals provided by the day-care service in our study represent an important additional resource to prevent anemia in these vulnerable groups.

The day-care staff and primary caregivers in this study showed a common understanding of the importance of including iron-rich ASF in the complementary feeding period to prevent anemia, as an important facilitator. They also showed awareness of national recommendations for complementary feeding and adapted them according to their daily experiences with children. This is consistent with the periodic training that the day-care staff receive to reinforce their knowledge and skills in anemia prevention topics as well as on children’s growth and development in Peru [21,29]. Studies in other LMICs have also reported that maternal counseling and antenatal education were positively associated with the intake of iron-rich foods in Ethiopia, highlighting the importance of counseling and strategies to increase the consumption of iron-rich foods during this critical stage of growth and development [30,31]. Nevertheless, our study also reported the existence of fears and misconceptions around the consumption of iron-rich ASFs among primary caregivers. Cultural norms and religious beliefs represent a barrier to be addressed in these settings by multiple actors and institutions. Future standard and national training should focus more on promotion of responsive feeding practices, an important component of complementary feeding recommendations, particularly around the consumption of iron-rich ASF and iron supplement rejection [32], as well as other culturally appropriate behavior change strategies such as positive deviation, to highlight the benefits of these foods to primary caregivers of IYC.

Our findings showed that inter-institutional coordination was an important facilitator for the day-care strategies. The linkages between institutions enabled communication and interaction between the day-care facilities, healthcare centers, and households of children under 2 years. This articulated system provided a strong support network for primary caregivers to ensure that they attended their local health center for child growth and development monitoring checks, as well as transferring the practices of iron supplementation and consumption of iron-rich ASFs from the day-care center to the household. This inter-institutional strategy enhanced the implementation of the current national policy to prevent anemia in children under 5 years [5], supported by the district technical manager and mother guides.

The close coordination of different government and non-governmental organizations (NGOs) was successfully achieved in Peru previously to tackle stunting and combat malnutrition [33]. During the 1990s, Peru was a leading example of partnerships between governmental and non-governmental actors to overcome malnutrition in the form of stunting [34]. Similar multi-faceted strategies and partnerships, in addition to the evidence-based implementation of programs, may also be a leading example regarding efforts to tackle anemia through day-care and household level programs.

In the day-care facilities, the nursery caregivers are close to the children and have a key involvement in their daily activities to, ultimately, achieve the goals of the national program. The nursery caregivers and mother guides are volunteers from the local communities who are identified and proposed by a network of community leaders. Therefore, these actors are trusted in the community and even though they may lack formal higher education, they are hired and then trained by the technical team, contributing to their recognition in the community. However, previous evidence reported that the Cuna Mas program experienced difficulties in recruiting and retaining child care staff [13,35], since these are volunteers who only receive a small financial incentive rather than a full living wage [36]. Regardless of this limitation, the day-care staff showed strong commitment and enthusiasm for their work, peer feedback, recognition and significant self-learning. This is an important role that could be considered for day-care programs more widely.

Whilst the day-care complementary feeding strategy provides a clear social behavioral change experience to ensure the consumption of iron-rich ASF and iron supplementation in day-care services for 5 days a week, this remained a challenge for the primary caregiver to implement at home. Therefore, further integrated strategies should consider secure access to, and availability of, iron-rich ASF in households with children under 2 years old in addition to the cooking demonstrations that showed a positive impact. The process of rejection, adaptation and acceptance to meals learnt through the daily experiences of these nursery caregivers, as well as the accompaniment by day-care staff, can be shared and used to show primary caregivers and health personnel that children learn to accept these meals and supplements.

The importance of the Cuna Mas work has been previously reported in 2017 in an impact evaluation report of the day-care service [13]. These results showed a positive effect on the development of children attending the day-care centers, assessing aspects such as effective verbal communication and emotion regulation. Likewise, another study in similar settings found that primary caregivers perceived a better nutritional status in their children due to the day-care service [37]. Another study found a significant reduction in the prevalence of anemia in a Peruvian Amazonian community due to the implementation of the articulated National Program to prevent and combat anemia, including the Cuna Mas services [16]. However, this research was conducted in a different geographical region than our current study and other factors may have influenced in their results.

To our knowledge, this is the first study that provides evidence of the facilitators and barriers on the nutritional practices to tackle anemia in the urban day-care service in Peru. The day-care centers represent an important intervention strategy which provides opportunities for mothers to seek employment, while supporting an optimal young child development and nutritional status. However, the urban day-care service is available to only a small proportion of low-income IYC in the country, targeting those in greatest poverty, it requires considerable funding and it relies heavily on volunteer workers and community care.

### Strengths and limitations

Our study has several strengths. The acceptance rate to participate was 100%, which allowed us to include the multi-actor perspectives in our analyses. Our design included two different urban settings in the Peruvian coastal and Andean regions which allowed us to explore potentially different challenges and contexts. While most findings were similar in the two study sites, primary caregivers in Huanuco city tended to report more challenges in the accessibility of iron-rich ASF than their counterparts in Lima. Furthermore, the qualification of the interviewers and expertise in qualitative interviews facilitated entry to these communities and in-depth data collection in both study sites.

The data triangulation from different methodologies, as well as input from the inter-disciplinary investigators, led to identification of the different perspectives of actors and a better understanding of the anemia prevention strategies in the day-care facilities. Likewise, the trust built with the actors during the direct observations made them feel more comfortable expressing their perspectives during the interview.

However, our study encountered some limitations. The study was affected by the onset of the COVID-19 pandemic lockdown in the country, and we could not conduct direct observations at the primary caregivers’ households as originally planned. The timeframe for direct observations in each day-care center was limited to 1 day while 3 days are usually recommended in order to capture variations in the daily routine. Instead of conducting repeated observations in the same centers, we opted to include 1 day of observations across multiple day-care centers to enrich the diversity of observations across day-care centers and study sites. A further potential limitation is that we did not use a standardized data collection template for observations, rather rapid observation notes were used and written up as reports later. This could have led to some variations between observers.

Furthermore, our study did not assess the entire Cuna Mas day-care service coverage in the study areas, which may help to inform the uptake and use of these services by the target population. As this was a qualitative study, our study was not designed to assess the impact of nutritional strategies within day-care centers on the anemia status of participating IYC. Evaluation of the impact of day-care programs on anemia would require a longitudinal design with a nationally representative urban sample which was beyond the scope of this study.

### Policy implications

The complementary feeding service at the day-care facilities represents an important example of anemia prevention in children through iron-rich ASF and supplements intake. Policy makers or other stakeholders could formalize the day-care staff role through merit recognition of this workforce, which plays a critical role in childhood health and well-being at the day-care services. Further improvements to the program could focus on addressing some of the perceptions, fears and misconceptions among primary caregivers on the prevention of iron deficiency anemia, as well as strengthening the supervision and counseling role of the nursery caregiver volunteers and the mother guides. The articulation between the health service and national day-care program enhances the implementation of the strategies to combat anemia in the population attending the day-care service, aligned with national policies. These insights are useful for similar programs and settings addressing these concerns, especially in developing novel and practical strategies to address this prevalent health problem in young children.

## Conclusions

The day-care program adapted national recommendations to promote the consumption of iron-rich ASF and iron supplements. The day-care staff and primary caregivers had a common understanding about the importance of preventing anemia in IYC which was underpinned by the practices at the day-care services and recommendations for the household.

The complementary feeding program with iron-rich ASF and iron supplements implementation involved applying diverse standardized and personalized strategies that were shared with primary caregivers. These institutional behavioral change initiatives could be replicated in other settings whilst considering the facilitators and barriers identified in this study.

## Data Availability

The direct observation and interview guides, codebooks for analysis and data that support the findings will be made available as open access resources via the Loughborough University Research Repository for the PERUSANO study (10.17028/rd.lboro.21558018, 10.17028/rd.lboro.21558033 and 10.17028/rd.lboro.21558048) following a period of embargo.
